# Navigating the Complexities: A Rare Case of Intrahepatic Cholestasis of Pregnancy With Placenta Previa Manifesting With Seizures

**DOI:** 10.7759/cureus.67385

**Published:** 2024-08-21

**Authors:** Pankaj Salvi, Ayushi Bhadoriya, Vidya Gaikwad, Himali Hatwar, Sneha Aramandla, Ashton Dsouza

**Affiliations:** 1 Obstetrics and Gynaecology, Dr. D. Y. Patil Medical College, Hospital and Research Centre, Dr. D. Y. Patil Vidyapeeth (Deemed to be University), Pune, IND

**Keywords:** feto maternal morbidity, antenatal care, intrahepatic cholestasis of pregnancy (icp), seizures, obstetrics and gynaecology

## Abstract

Intrahepatic cholestasis of pregnancy (ICP) is a prevalent and reversible liver disorder that occurs during pregnancy. It is primarily characterized by itching, especially on the palms and soles, and elevated levels of transaminases and bile acids. Some patients may also exhibit hyperbilirubinemia. This condition generally has a good maternal prognosis. The patient, in this case, presented with severe itching, elevated liver enzymes and bile acids, and an ultrasound indicated placenta previa. Uniquely, she experienced an episode of seizure and high blood pressure following surgery. This case report underscores the need for vigilant monitoring of patients with ICP, not only during pregnancy due to the risk of adverse perinatal outcomes but also for antenatal and postpartum complications.

## Introduction

Intrahepatic cholestasis of pregnancy (ICP), also known as icterus gravidarum, is a prevalent liver condition during pregnancy characterized by intense itching and sometimes jaundice. The incidence and prevalence of ICP vary by ethnicity and geographic location, with rates ranging from 0.2% to 2% of pregnancies [1]. ICP frequently occurs in familial clusters, indicating a significant hereditary component, as supported by its increased occurrence in certain areas [2]. The pathophysiology is significantly influenced by mutations in hepatobiliary transport proteins, such as the multidrug resistance protein 3 (MDR3), which is implicated in the biliary secretion of phospholipids. Mutations in genes such as the multidrug resistance protein 2 (MDR2) and bile salt export pump (BSEP) can result in ICP or drug-induced liver damage [3]. Although the exact role of sex steroids is unknown, the cholestatic effects of progesterone sulphate metabolites and 17-β-D-oestradiol are well established [4].

Severe itching, which usually worsens at night and is particularly intense on the palms and soles, is one of the symptoms of ICP. Additional symptoms may include upper right quadrant pain, nausea, fatigue, loss of appetite, dark urine, and pale stools [5]. Perinatal outcomes linked to ICP include preterm birth, meconium-stained amniotic fluid, abrupt intrauterine death, and neonatal Intensive Care Unit (ICU) admissions. Elevated levels of liver enzymes, such as aspartate transaminase (AST) and alanine transaminase (ALT), do not correlate with pregnancy outcomes [6].

According to the Society for Maternal and Fetal Medicine, the diagnosis of ICP involves determining the presence of pruritus symptoms, verifying elevated levels of total serum bile acids, and ruling out other conditions that may present with similar laboratory findings and symptoms. A total serum bile acid level greater than 10 mmol/L is typically required for the diagnosis of ICP. Although elevated transaminase values, such as those of ALT and AST, can be observed in ICP, they are not necessary for diagnosis [7].

Although ICP generally has a good prognosis and is reversible post-delivery, it can be associated with seizures and may become severe. We report a case of a second gravida with ICP who presented with seizures postoperatively.

## Case presentation

A 28-year-old female, second gravida at 34 weeks of gestation, presented with complaints of intermittent abdominal pain for two to three days and intense itching on the palms and soles for three to four days. Her history included a lower segment caesarean section (LSCS) performed three years ago due to fetal distress. She had no significant history of ICP in her previous pregnancy or any seizure disorders.

On general examination, her vitals were stable, with no signs of icterus, and the systemic examination was within normal limits. On abdominal examination, the uterus corresponded to 34 weeks of gestation and was relaxed on palpation, with a previous caesarean scar noted, but without signs of tenderness. On per vaginal examination, her cervical os was closed.

**Figure 1:**
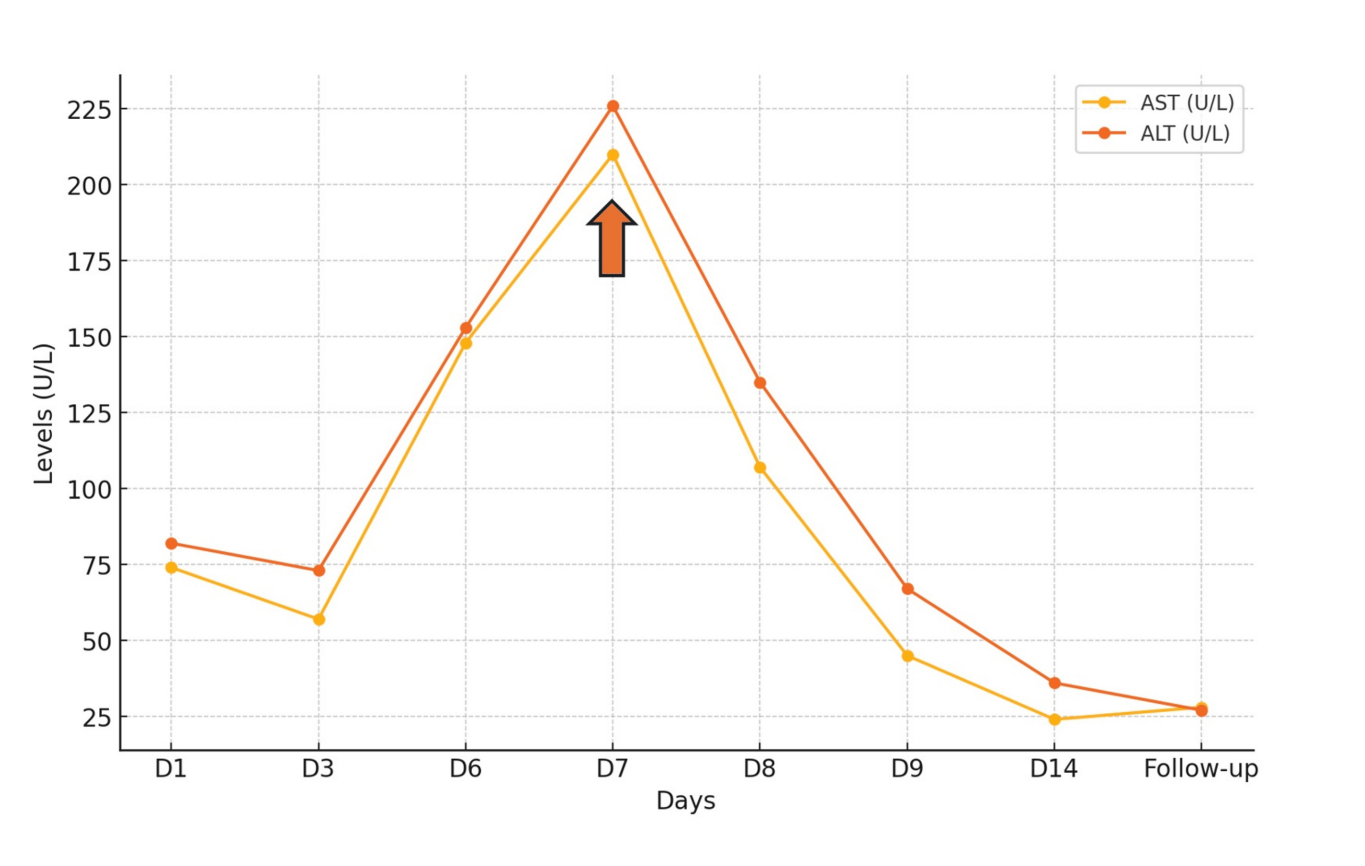
AST and ALT levels over time (throughout admission) with the arrow indicating peak values on day 7 of admission D: Day; AST: Aspartate transaminase; ALT: Alanine transaminase

**Figure 2:**
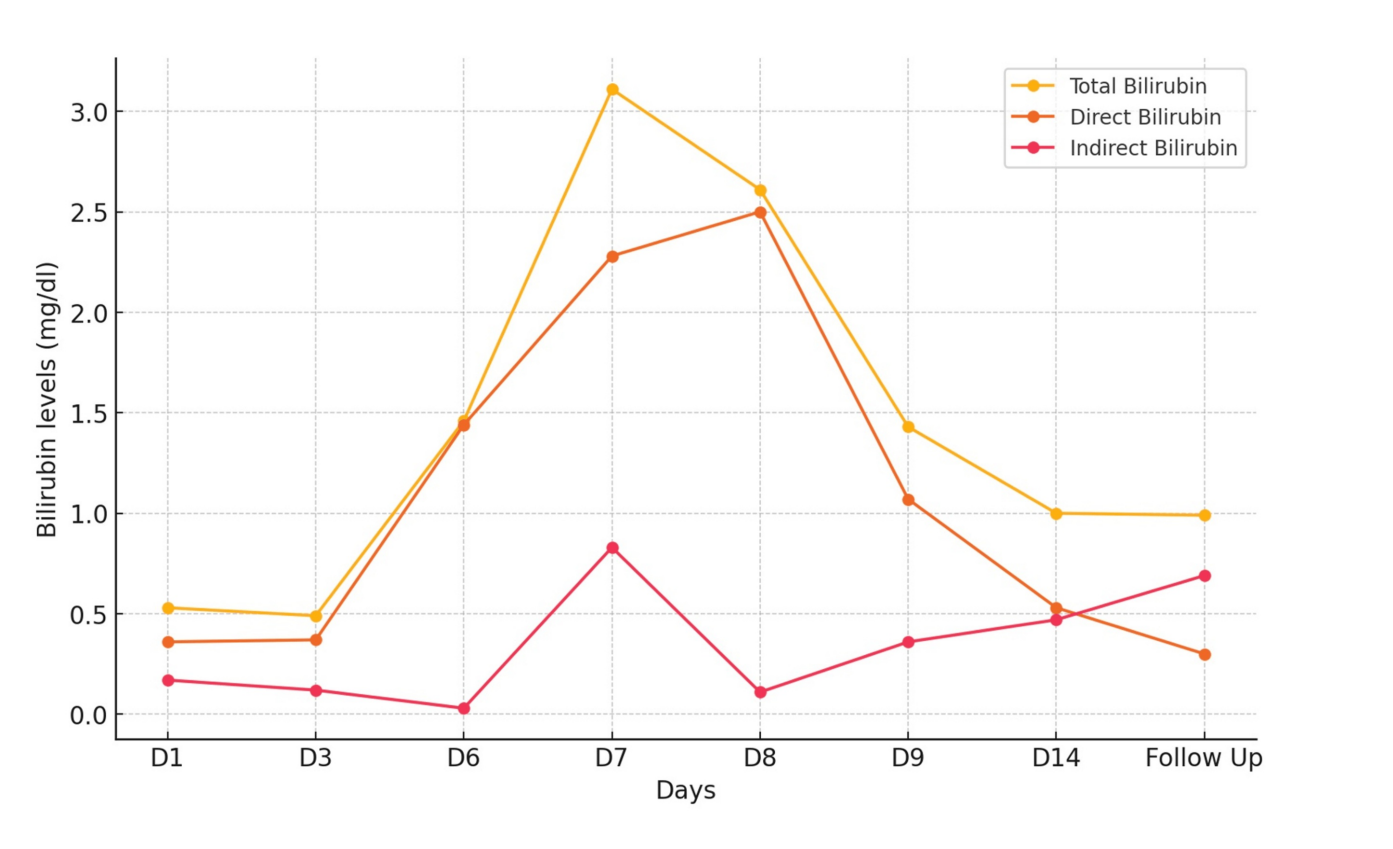
Bilirubin levels measured over time (throughout admission) D: Day

Routine laboratory investigations, including a complete blood count, liver enzymes, serum bile acids, and coagulation profile, were conducted upon admission and monitored throughout her stay (Table 1). The laboratory reports confirmed a diagnosis of ICP, as her liver enzymes were elevated, with AST at 74 U/L, ALT at 82 U/L, and serum bile acids at 58 µmol/L (Table 1). Her AST, ALT, total bilirubin, and direct and indirect bilirubin levels were measured throughout her admission to rule out other liver disorders (Figures 1-2). Hepatitis serology was negative for infective hepatitis. A routine urine examination was negative for bile salts and pigments. The patient was given corticosteroids and started on a tablet of ursodeoxycholic acid (UDCA) 300 mg, three times a day.

**Table 1:** Laboratory investigations throughout admission D: Day; LSCS: Lower segment caesarean section; ALT: Alanine transaminases; AST: Aspartate transaminases; BSL-R: Blood sugar random; LDH: Lactate dehydrogenase; GGT: Gamma-glutamyl transferase; PT: Prothrombin time; INR: International normalized ratio; aPTT: Activated partial thromboplastin time

Blood investigations	D1	D3	D6	D7 (day of LSCS)	D8	D9	D14	Follow up after one week	Normal values
Haemoglobin (g/dL)	13.7	12.8	12.5	13.2	12	12.2	12.1	12.8	11-15 g/dL
Platelets (/μL)	2,11,000	1,76,000	1,55,000	1,51,000	98,000	1,05,000	1,89,000	1,90,000	150,000-400,000 /μL
AST (U/L)	74	57	148	210	107	45	24	28	8-43 U/L
ALT (U/L)	82	73	153	226	135	67	36	27	7-45 U/L
Total bilirubin (mg/dL)	0.53	0.49	1.46	3.11	2.61	1.43	1	0.99	0.2-1.2 mg/dL
Direct bilirubin (mg/dL)	0.36	0.37	1.44	2.28	2.5	1.07	0.53	0.30	Upto 0.5 mg/dL
Indirect bilirubin (m/dL)	0.17	0.12	0.03	0.83	0.11	0.36	0.47	0.69	0.12-1.0 mg/dL
Serum LDH (U/L)	236	234	281	320	-	494	343	-	81-234 U/L
Serum GGT (U/L)	81	77	114	148	122	96	115	-	5-36 U/L
Serum bile acids (mmol/L)	58	-	>200	-	45.25	68.26	3.75	-	<10 mmol/L
BSL-R (mg/dL)	90	110	87	90	112	98	86	-	<200 mg/dL
PT (sec)	10.4	10.4	-	-	11.7	-	10.1	-	10.24-12.71 sec
INR (sec)	0.8	0.8	-	-	0.9	-	0.8	-	0.85-1.15 sec
aPTT (sec)	25.9	22.8	-	29.20	27.4	-	32.30	-	24.71-34.3 sec
D-dimer (ng/mL)	2284	2342	-	5716	3108	-	2144	-	0-500 ng/mL
Serum fibrinogen (mg/dL)	433	322	-	222	215	-	426	-	238-498 mg/dL
Serum uric acid (mg/dL)	8.2	8.4	8.10	-	11.60	8.4	5.7	-	2.7-6.1 mg/dL

An obstetric ultrasound with Doppler was performed, revealing a single live, healthy foetus with grade 3 placenta previa (posterior) and no significant Doppler findings. The patient was instructed to keep a daily fetal movement kick count, and a non-stress test was performed daily for antepartum fetal surveillance. Repeat laboratory investigations during admission showed a significant rise in liver enzymes on the third day compared to previous values (Table 1). Given the increasing trend of liver enzymes, reaching peak values on day 7 (Figure 1), and serum bile acids >200 µmol/L, a decision was made to terminate the pregnancy. She was given 10 mg of vitamin K intramuscularly and then taken for an emergency LSCS due to the previous LSCS, placenta previa, and ICP. She delivered a healthy male baby weighing 2.5 kg. Due to excessive oozing, bilateral uterine artery and anterior division of the internal iliac artery ligation were performed. The patient received two pints of fresh frozen plasma (FFP) (1 pint = 250 mL FFP) preoperatively and two pints intraoperatively to prevent the possible complication of epidural hematoma. She was given 600 micrograms of misoprostol sublingually postoperatively. The procedure was uneventful.

**Figure 3:**
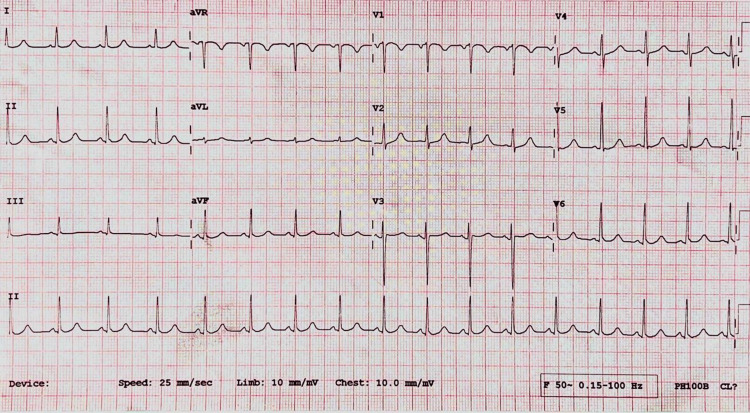
ECG suggestive of sinus tachycardia ECG: Electrocardiogram

After being shifted to the ICU for observation, the patient developed a generalized tonic-clonic seizure lasting one minute. She experienced postictal confusion; her vitals showed a blood pressure of 160/100 mmHg and a pulse rate of 140 beats per minute. Her electrocardiogram (ECG) indicated sinus tachycardia (Figure 3). The urine dipstick test was negative for urine albumin. Arterial blood gas (ABG) analysis suggested metabolic acidosis with respiratory acidosis and lactic acidosis, as shown in Table 2.

**Table 2:** Arterial blood gas analysis (post-operatively)

Test	Value	Normal range
pH	7.1	7.35-7.45
pCO_2_ (mm of Hg)	58	35-45
pO_2 _(mm of Hg)	60	65-105
Bicarbonate (HCO_3_^-^) (mEq/L)	12	22-26
Lactate (mmol/L)	8	<2

The patient was stabilized with 1 g of levetiracetam administered intravenously and then mechanically ventilated. Suspecting eclampsia, we administered an injection of magnesium sulphate with a loading dose of 4 g intravenously, followed by 5 g intramuscularly in each buttock. She was started on a labetalol infusion at 10 mL/hour for six hours to control her blood pressure. Neurophysicians were consulted to rule out cerebral venous sinus thrombosis and hepatic encephalopathy. Magnetic resonance imaging (MRI) with venogram was advised, along with tests for serum calcium, magnesium, phosphate, and serum ammonia, all of which were normal (Table 3). The patient was started on levetiracetam 500 mg twice daily, as advised.

**Table 3:** Laboratory values (post-operatively)

Parameters	Values	Normal range
Serum calcium (mg/dL)	10.6	8.6-10.2
Serum magnesium (mg/dL)	2.3	1.8-2.4
Serum ammonia (mg/dL)	43	20-120

A gastroenterology consultation advised continuing UDCA 300 mg twice daily, adding rifaximin 550 mg twice daily, and syrup lactulose 30 mL once daily. The MRI report was normal, but the ultrasound revealed a prominent common bile duct and minimal free fluid in Morrison's pouch. The patient was extubated 24 hours later, monitored closely, and shifted to the ward three days later. Her sutures were removed six days later, and she was discharged on the 14th day, when her laboratory parameters returned to normal (Table 1). The patient was asked to follow up one week later with liver function test (LFT) reports.

## Discussion

Intrahepatic cholestasis is one of the most prevalent liver diseases during pregnancy, characterized by elevated liver enzymes and serum bile acids, along with pruritus. While the prognosis for mothers with this reversible condition is generally good, complications may occur if the patient presents with seizures and postpartum haemorrhage (PPH). Pruritus is a common symptom that may arise from various physiological, clinical, or inflammatory conditions. Because intrahepatic cholestasis can negatively impact pregnancy outcomes, a woman presenting with pruritus must be treated seriously [8].

Compared to a normal pregnancy, serum alkaline phosphatase levels are higher in ICP, and while elevated aminotransferases may be present, they are not diagnostic. ICP is diagnosed when total serum bile acid levels exceed 10 mmol/L [9]. ICP is significant because it has been linked to several unfavourable perinatal outcomes, such as preterm birth, stillbirth, and meconium-stained amniotic fluid [7]. These cholestatic effects may cause alterations in the composition of bile, favouring the predominance of hydrophobic acids by altering the ratio of hydrophilic to hydrophobic bile acids. This can impair the ability of water-soluble bile acids to pass through the placenta and be eliminated by the mother's kidneys. In a pregnancy affected by intrahepatic cholestasis, this transplacental transfer is reversed, moving from the mother to the foetus [10].

Given that it lowers bile acid levels and is well tolerated by patients, UDCA is recommended as the first-line treatment for maternal ICP symptoms. A dose of 300 mg should be administered two to three times a day. Additional drugs, such as rifampin, cholestyramine, and S-adenosyl-L-methionine, can be utilized for patients who do not respond to UDCA or are refractory to it [7]. S-adenosyl-methionine is less effective than UDCA in relieving pruritus. Cholestyramine binds to bile acids in the intestine and prevents their reabsorption, which reduces bile acid reabsorption, decreases gastrointestinal side effects, such as nausea and constipation, and improves liver function, though it does not alleviate pruritus associated with ICP. Combining rifampin with UDCA has been found to reduce pruritus in cases of refractory ICP by improving bile acid detoxification, providing a complementary effect to the up-regulation of bile acid export induced by UDCA [11].

While the timing of delivery is tailored to each patient, many authors support the overarching concept of weighing the risks of unexpected intrauterine fetal death against those of premature delivery before determining when to deliver the baby. All women who are less than 34 weeks of gestation should receive antenatal corticosteroids. According to the American College of Obstetricians and Gynecologists (ACOG), pregnancies complicated by ICP should be delivered between 36+0 and 37+0 weeks of gestation. Concerning indicators include a history of intrauterine fetal death before 37 weeks due to ICP, a total serum bile acid concentration exceeding 100 µmol/L, and maternal symptoms like jaundice that are unresponsive to medication and require increasing doses of UDCA. In such cases, the patient should be delivered before 37 weeks of gestation [12].

This condition affects biliary function, leading to fat malabsorption, poor vitamin absorption, and potentially a vitamin K deficiency, which may result in severe PPH associated with ICP [13]. Hence, when our patient was taken for a caesarean section, she was already at high risk of PPH due to placenta previa, compounded by the added complication of ICP. Her estimated blood loss exceeded 1000 mL, and there was persistent oozing, which necessitated the ligation of both uterine arteries and the anterior division of the internal iliac arteries to achieve haemostasis. Her medical complications had not yet fully resolved, as she presented with seizures one hour later in the ICU.

Our patient exhibited no signs of gestational hypertension or pre-eclampsia throughout her entire antenatal period. However, she developed generalized tonic-clonic seizures, and her blood pressure reached 160/100 mmHg. The possibility of atypical eclampsia was considered, despite the absence of proteinuria, as she did not exhibit the typical signs of pre-eclampsia or eclampsia [14]. Another potential cause of the convulsions could be fever, shivering, and central nervous system (CNS) excitation following the use of misoprostol, which was administered to treat PPH and prevent secondary PPH. This suspicion is supported by another case report that documented misoprostol-induced convulsions as a rare adverse effect [15].

In pregnancy, the management of epilepsy requires the judicious selection and monitoring of antiepileptic drugs, as certain medications may increase the risk of teratogenic effects. At the same time, uncontrolled seizures pose significant risks to both maternal and fetal health. Both levetiracetam and lamotrigine are regarded as safe antiepileptic drugs for use during pregnancy, with lamotrigine being considered safer due to its lower risk of causing major congenital malformations [16]. In our patient, since she had a seizure postoperatively, we opted to administer 1 g of intravenous levetiracetam, given its high therapeutic index and suitability for continued prophylactic use, as it remains effective when taken orally.

ICP has been linked to severe pre-eclampsia and eclampsia in several studies. For instance, Granese et al. observed that patients with ICP had a statistically significant increase in the risk of developing conditions such as gestational diabetes, gestational hypertension, hypothyroidism, and thrombophilia compared to individuals without ICP [17]. Another study by Avsar et al. supports this finding, showing that women with ICP have a higher risk of preeclampsia than women without the condition [18].

Endothelial dysfunction shares the same pathophysiology as pre-eclampsia and eclampsia and can be triggered by increased oxidative stress, damage to endothelial cells from disrupted bile acid regulation, and subsequent inflammatory responses in ICP [19]. The patient's clinical presentation led us to consider another differential diagnosis: normotensive HELLP (Haemolysis, Elevated Liver Enzymes, Low Platelets) syndrome. She exhibited low platelets and elevated liver enzymes, with increased serum lactate dehydrogenase (LDH). However, there were no signs of haemolysis on the peripheral smear, and her indirect bilirubin levels were not elevated, though her total serum bilirubin levels were. Additionally, she did not have hypertension or proteinuria [20]. Similar cases were reported by Jebbink et al., who discussed four patients with ICP complicated by HELLP syndrome during pregnancy [21].

This case report highlights a rare, complex presentation of ICP with grade 3 placenta previa, manifesting with seizures postoperatively, which could be due to normotensive HELLP syndrome or drug-induced causes. Obstetricians need to be vigilant about the wide range of potential complications associated with ICP, and further studies are needed to evaluate maternal complications related to this disorder.

## Conclusions

This case highlights the complex presentation of intrahepatic cholestasis and the multidisciplinary management required for a pregnant patient with grade 3 placenta previa and ICP, who manifested with seizures postoperatively. These seizures could potentially be attributed to normotensive HELLP syndrome or drug-induced factors. It is critical to take pruritus in pregnant patients seriously and ensure that serum bile acids are tested promptly. ICP can significantly impact both perinatal and maternal outcomes, leading to potential severe morbidity and mortality. Early diagnosis and comprehensive management are essential to mitigate these risks.
